# Assessment of a 96-Well Plate Assay of Quantitative Drug Susceptibility Testing for Mycobacterium Tuberculosis Complex in China

**DOI:** 10.1371/journal.pone.0169413

**Published:** 2017-01-12

**Authors:** Hui Xia, Yang Zheng, Bing Zhao, Susan van den Hof, Frank Cobelens, YanLin Zhao

**Affiliations:** 1 National Tuberculosis Reference Laboratory, National Center for Tuberculosis Control and Prevention, Chinese Center for Disease Control and Prevention, BeiJing, People’s Republic of China; 2 KNCV Tuberculosis Foundation, The Hague, Netherlands; 3 Faculty of Medicine of the University of Amsterdam, Amsterdam, Netherlands; The University of Hong Kong, CHINA

## Abstract

**Objective:**

To evaluate the performance of the Sensitire MYCOTB MIC Plate (MYCOTB) which could measure the twelve anti-tuberculosis drugs susceptibility on one 96-wells plate.

**Methods:**

A total of 140 MDR-TB strains and 60 non-MDR strains were sub-cultured and 193 strains were finally tested for drug resistance using MYCOTB and agar proportion method (APM) and another 7 strains failed of subculture. The drugs included ofloxacin (Ofx), moxifloxacin (Mfx), rifampin (RFP), amikacin (Am), rifabutin (Rfb), para-aminosalicylic acid (PAS), ethionamide (Eth), isoniazid (INH), kanamycin (Km), ethambutol (EMB), streptomycin (Sm), and cycloserine(Cs). The categorical agreement, conditional agreement, sensitivity and specificity of MYCOTB were assessed in comparison with APM. For strains with inconsistent results between MYCOTB and APM, the drug resistance related gene fragments were amplified and sequenced: *gyrA* for Ofx and Mfx; *rpoB* for RFP and Rfb; *embB* for EMB; *rpsl* for Sm; *katG* and the promoter region of *inhA* for *INH*, *ethA* and the promoter region of *inhA for* Eth. The sequence results were compared with results of MYCOTB and APM to analyze the consistency between sequence results and MYCOTB or APM.

**Results:**

The categorical agreement between two methods for each drug ranged from 88.6% to 100%. It was the lowest for INH (88.6%). The sensitivity and specificity of MYCOTB ranged from 71.4% to 100% and 84.3% to 100%, respectively. The sensitivity was lowest for Cs(71.4%), EMB at 10μg/ml (80.0%) and INH at 10.0μg/ml (84.6%). The specificity was lowest for Rfb (84.3%). Overall discordance between the two phenotypic methods was observed for 96 strains, of which 63 (65.6%) were found susceptible with APM and resistant with MYCOTB and the remaining 33(34.4%) strains were resistant by APM and susceptible with MYCOTB. 34/52 (65.4%) sequenced APM susceptible and MYCOTB resistant(APM-S/MYCOTB-R) strains had mutations or insertions in the amplified regions. 20/30 (66.7%) sequenced APM resistant and MYCOTB susceptible strains had mutations in the sequenced genes. MICs of twenty-nine of these thirty isolates were equal to or within 1 doubling dilution of the critical concentration.

**Conclusion:**

MYCOTB had good performance for most of tested drugs and could be used as an alternative to the more labor demanding and longer turnaround time solid culture based DST method for detection of drug susceptibility in China.

## Introduction

TB has become a global challenge due to the emergence of multi-drug resistant tuberculosis (MDR-TB) and extensively drug-resistant tuberculosis (XDR-TB). A national drug resistance survey done in China in 2007 showed that 5.7% of new and 25.6% of previously treated TB cases were MDR, and 0.5% and 2.1% respectively were XDR[1]. Early and accurate detection of drug resistance is one of the priorities of TB control programs. Solid culture based proportion DST method were widely used in china, however, which will take at least 4–6 weeks to report the result. The World Health Organization (WHO) recently recommends using the cartridge-based real-time PCR Xpert MTB/RIF assay for detecting pulmonary tuberculosis and rifampin resistance, and the GenoType MTBDRplus line-probe assay for rapid detection of rifampin resistance and isoniazid resistance. A drawback of such molecular methods is that they include only some of the first line anti-tuberculosis drugs and does not provide susceptibility of other anti-tuberculosis drugs used for clinicians to make appropriate choices to give individual regimen of anti-tuberculosis treatment and in particular with regard to second-line drugs used in the treatment of MDR and XDR-TB. Furthermore, the molecular based diagnostic tools detect only some gene mutations in the hot-spot region associated with drug resistance and may not cover all mutations associated with resistance. For instance, single nucleotide polymorphisms (SNPs) outside the rifampin resistance determining region (RRDR) near the beginning of the *rpoB* gene have been described to be associated with rifampin resistance and molecular assays that only target the RRDR in the *rpoB* gene may give false susceptible result[2]. For some drugs, conventional DST methods did not reflect the resistance very well compared with new methods. One study reported that there were a number of ofloxacin-susceptible strains by conventional methods but resistant by MYCOTB and genotypic methods (*gyrA* mutant) [3]. Another study conducted by Sayera et al showed that DST of streptomycin worked well with MYCOTB but less well for the conventional phenotypic methods[4]^.^

The clinician sometimes may use higher dose of medicines for patients with isolated strains with quantitative result indicating a borderline susceptible as for the fluoroquinolone [4].MIC result is very important for individualized patient care as it gives a quantitative result that may inform potential dose increases or within-class changes[5]. The MYCOTB plate (MYCOTB; Trek Diagnostic Systems, Thermo Fisher Scientific, United States) is a 96 well microtiter plate to which the manufacturer has added twelve lyophilized antibiotics and can detect resistance at various MIC (minimal inhibition concentration) levels and determine borderline susceptibility rather than at a defined critical concentration to give a categorized resistance or susceptibility. In the following study we evaluated the performance of MYCOTB in China.

## Materials and Methods

### Strains

A total of 140 MDR-TB strains isolated during national drug resistance survey in 2007 and 60 non-MDR strains with other drug resistance patterns preserved in National Reference Laboratory were recovered on Löwenstein-Jensen medium (L-J medium). 193 strains were finally tested using the 7H10 Agar Proportion Method (APM) and the MYCOTB assay.

### Drug susceptibility testing with agar proportion method

7H10 agar media containing critical concentration (CC) drugs were prepared in house according to the Clinical and Laboratory Standards Institute (CLSI) methods for indirect testing of susceptibility of *Mycobacterium tuberculosis* to anti-tuberculosis drugs. Critical concentrations for each drug were as follows: 2.0μg/ml for ofloxacin (Ofx), 0.5μg/ml and 2.0μg/ml for moxifloxacin (Mfx), 1.0μg/ml for rifampin (RFP), 4.0μg/ml for amikacin (Am), 0.5μg/ml for rifabutin (Rfb), 2.0μg/ml for para-aminosalicylic (PAS,) 5.0μg/ml for ethionamide (Eth), 0.2μg/ml and 1.0μg/ml for isoniazid (INH), 5.0μg/ml for kanamycin (Km), 5.0μg/ml and 10.0μg/ml for ethambutol (EMB), 2.0μg/ml and 10.0μg/ml for streptomycin (Sm) and 25.0μg/ml for cycloserine (Cs). All media were used within 2 weeks of preparation. Agar plates were incubated at 37°C for 3 weeks before interpretating results[6].

### Drug susceptibility testing with MYCOTB

MYCOTB tests were performed according to the manufacturer’s instructions. A loop of freshly grown bacilli were scraped from the surface of L-J medium and added to saline-tween-80 solution with glass beads, homogenized through vertex and adjusted to a 0.5 McFarland standard using the Sensititre Nephelometer. 100ul suspension was transferred to 11 ml of Middlebrook 7H9 broth supplemented with oleic acid-albumin-dextrose-catalase (OADC). Vortex for 30 seconds. The control agar medium with inoculum of 10^−4^ dilution of standardized strain suspension when conducting drug susceptibility testing by agar proportion method can give countable colonies and verified that the bacterial concentration was within the targeted amount(∼10^5^ CFU/ml). 100ul bacterial suspension was transferred to the MYCOTB plate wells containing the antibiotics. Plate was covered with the seal and incubated at 37°C and monitored by specified plate reader at days 10 after inoculation. If the growth of control well is poor after 10 days incubation, the plate will be incubated again for additional 4 days or 11 days until the growth control can be visible. The suspensions in all wells were checked after 24 hours and 48hours incubation and before reading results at the end of incubation for contamination. The MIC were determined only when all wells in plates were clear and not contaminated. For each drug, the lowest concentration with no visible growth was considered to be the MIC.

### Sequencing

For strains with inconsistent susceptibility results between MYCOTB and APM, two methods were firstly repeated and if the results were different between the initial and repeated tests, the test was repeated again. For strains with inconsistent results after repeats, we amplified the drug resistance related gene fragments and sequenced them by the methods reported previously [7,8,9,10] for Ofx, Mfx, RFP, Rfb, EMB, Sm, INH and Eth. The sequencing was not conducted for strains with discrepant results for Cs and PAS since the mechanisms of resistance for these two drugs were not as clear as for other drugs. For drugs with two critical concentrations, we only calculated the low level concentration for analyzing the sequencing results for strains with discrepant susceptibility results for Mfx, EMB, Sm. The discrepant strains by two methods for INH at high level concentration (1.0μg/ml) were also sequenced. The gene fragments sequenced included *gyrA* for Ofx and Mfx; *rpoB* for RFP and Rfb; *embB* for EMB; *rpsl* for Sm; *katG* and the promoter region of *inhA*, *ethA* and the promoter region of *inhA* for Eth (Table 1). The sequencing results were entered into the Basic Local Alignment Search Tool (BLAST, www.ncbi.nlm.nih.gov/BLAST) for comparison with the corresponding genes of the *M*. *tuberculosis* strain H37Rv.

**Table 1:** Oligonucleotide primers for sequencing of the genes associated with drug resistance.

Gene	Primer name	Sequence (5'-3')	Amplicon size (bp)
*rpoB*	rpoB-1f	CTTGCACGAGGGTCAGACCA	543
	rpoB-2r	ATCTCGTCGCTAACCACGCC	
*inhA*	inhA-1f	TGCCCAGAAAGGGATCCGTCATG	455
	inhA-2r	ATGAGGAATGCGTCCGCGGA	
*katG*	katG-5	AACGACGTCGAAACAGCGGC	455
	katG-6	GCGAACTCGTCGGCCAATTC	
*embB*	embB-F	CTGACCGACGCCGTGGTGATAT	490
	embB-R	TGAATGCGGCGGTAACGACG	
*rpsl*	rpsL-F	GGCATGGCCGACAAACAGAACG	501
	rpsL-R	ACTGGGTGACCAACTGCGATCC	
*gyrA*	gyrA-F	CCCTGCGTTCGATTGCAAAC	423
	gyrA-R	CTTCGGTGTACCTCATCGCC	
*ethA*	ethA1	ATC ATC GTC GTC TGA CTA TGG	667
	ethA5	ACT ACA ACC CCT GGG ACC	
	ethA4	CCT CGA CCT TCC CGT GA	692
	ethA9	CCT CGA GTA CGT CAA GAG CAC	
	ethA8	GGT GGA ACC GGA TAT GCC TG	342
	ethA10	CGT TGA CGG CCT CGA CAT TAC	

### Definitions

The definitions of categorical agreement were referenced as in the study conducted by Jongseok et al.[11]. Briefly, isolates were categorized as susceptible by the MYCOTB when the MIC was equal to or lower than the critical concentrations for the conventional APM and resistant if greater than the critical concentration in the APM. Categorical agreement was defined as both MYCOTB and APM results being characterized as susceptible or resistant. It was considered to be conditional agreement if APM characterized the isolates susceptible and the MYCOTB MIC was less than or equal to the APM critical concentration plus 1 doubling dilution or if APM characterized the isolates resistant and the MYCOTB MIC was equal to or higher than the APM critical concentration.

### Data analysis

In the analysis of the data, APM was used as the reference standard for calculating categorical agreement, conditional agreement, sensitivity and specificity of the MYCOTB assay for each drug. All of data were analyzed using Microsoft Excel.

## Results

### Drug susceptibility testing results with agar proportion method

Among 193 strains, the proportion of resistant isolates by drug were 11.9% for Ofx, 11.4% and 1.6% for Mfx at 0.5μg/ml and 2.0μg/ml, 69.9% for RFP, 4.7% for Am, 53.9% for Rfb, 5.7% for PAS, 15.0% for Eth, 78.2% and 67.4% for INH at 0.2μg/ml and 1.0μg/ml, 4.7%for Km, 19.7% and 2.6% for EMB at 5.0μg/ml and 10.0μg/ml, 48.7% and 35.2% for Sm at 2.0μg/ml and 10.0μg/ml, 3.6% for Cs. The proportion of MDR was 69.9%.

### Comparison of MYCOTB with agar proportion method

The categorical agreement, conditional agreement, sensitivity and specificity of MYCOTB for all drugs are shown in Table 2. The categorical agreements between APM and MYCOTB for each drug ranged from 88.6% to 100%. It was lowest for INH at 1.0μg/ml (88.6%). The agreement was greater than 95% for all drugs except Rfb (93.8%). The conditional agreements between two methods were from 93.8% to 100.0%. The sensitivity and specificity of MYCOTB ranged from 71.4% to 100% and 84.3% to 100%, respectively. The sensitivity for detection of resistance was lowest for Cs (71.4%), EMB at 10.0μg/ml (80.0%) and INH at 1.0μg/ml (84.6%). The specificity was lowest for Rfb (84.3%). The detailed performances are shown in Table 2.

**Table 2:** Performance of MYCOTB compared with APM.

Drug (APM CC)	MYCOTB	APM		%Sensitivity (95%CI)	%Specificity (95%CI)	% Categorical agreement	% Conditional agreement
		R	S				
Ofx (2.0μg/ml)	R	23	5	100.0 (85.2–100.0)	97.1 (93.3–99.0)	97.4	100.0
	S	0	165				
Mfx (0.5μg/ml)	R	21	7	95.5 (77.2–100.0)	95.9 (91.2–98.3)	95.9	96.9
	S	1	164				
Mfx (2.0μg/ml)	R	3	7	100.0 (29.2–100.0)	96.3 (92.6–98.5)	96.4	99.0
	S	0	183				
RFP (1.0μg/ml)	R	135	4	100.0 (97.3–100.0)	93.1 (83.3–98.1)	97.9	99.0
	S	0	54				
Am (5.0μg/ml)	R	9	0	100.0 (66.4–100.0)	100.0 (98.0–100.0)	100.0	100.0
	S	0	184				
Rfb (0.5μg/ml)	R	101	14	97.1 (91.8–99.4)	84.3 (75.0–91.1)	91.2	93.8
	S	3	75				
PAS (2.0μg/ml)	R	10	5	90.9 (58.7–99.8)	97.3 (93.7–99.1)	96.9	97.4
	S	1	177				
Eth (5.0μg/ml)	R	28	6	96.6 (82.2–99.9)	96.3 (92.2–98.7)	96.4	98.0
	S	1	158				
INH (0.2μg/ml)	R	151	0	100.0 (97.6–100.0)	100.0 (91.6–100.0)	100.0	100.0
	S	0	42				
INH (1.0μg/ml)	R	110	2	84.6 (77.2–90.3)	96.8 (89.0–99.6)	88.6	95.3
	S	20	61				
Km (5.0μg/ml)	R	9	0	100.0 (66.4–100.0)	100.0 (98.0–100.0)	100.0	100.0
	S	0	184				
EMB (5.0μg/ml)	R	38	11	100.0 (90.8–100.0)	92.9 (87.7–96.4)	94.3	98.4
	S	0	144				
EMB(10.0μg/ml)	R	4	11	80.0 (28.4–99.5)	94.1 (89.8–97.0)	93.8	99.0
	S	1	177				
Sm(2.0μg/ml)	R	89	3	94.7 (88.0–98.3)	97.0 (91.4–99.4)	95.9	97.4
	S	5	96				
Sm (10.0μg/ml)	R	66	6	97.1 (89.8–99.6)	95.2 (89.8–98.2)	95.9	96.9
	S	2	119				
Cs (25.0μg/ml)	R	5	6	71.4 (29.0–96.3)	96.8 (93.1–98.8)	95.9	97.4
	S	2	180				

Note: CC: critical concentration; R: resistant; S: susceptible.

### Sequencing results for strains with discrepancies between APM and MYCOTB

Overall discordance between the two phenotypic methods was observed for 96 strains, of which 63 (65.6%) were found susceptible with APM and resistant with MYCOTB and the remaining 33(34.4%) strains were resistant by APM and susceptible with MYCOTB. A total of 74 strains had discrepant results for Ofx, Mfx(0.5μg/ml), RFP, Rfb, Eth, EMB(5.0μg/ml), Sm(2.0μg/ml), Cs and PAS. 17.6% (13/74) strains were APM-resistant but MYCOTB-susceptible (APM-R/MYCOTB-S) and 82.4% (61/74) strains were APM-susceptible but MYCOTB-resistant (APM-S/MYCOTB-R). Of note were that twenty (90.9%) strains were APM-R/MYCOTB-S and two (9.1%) were APM-S/MYCOTB-R among 22 discrepant strains for INH at high level concentration (1μg/ml). DNA was amplified and sequenced from 82 strains with discrepant results for the following drugs: Ofx, Mfx, RFP, Rfb, Eth, EMB, Sm and INH except 14 strains with inconsistent result for Cs and PAS. Sequencing results for strains with discrepant results between the two methods are shown in Table 3.

**Table 3:** Sequencing result of strains with discrepant results between APM and MYCOTB.

Discrepant results per drug	No.strains	MICs (μg/ml)	Sequencing results	
Ofx (2.0μg/ml)			*gyrA*	
APM-S/MYCOTB-R	5	4–8		• 2 Asp94Gly (GAC-GGC) • 1 Ser91Pro (TCG-CCG) • 1 Ala90Val (GCG-GTG) • 1 wildtype
Mfx(0.5μg/ml)			*gyrA*	
APM-R/MYCOTB-S	1	0.5		• 1 Ala90Val (GCG-GTG)
APM-S/MYCOTB-R	7	1–4		• 2 Asp94Gly (GAC-GGC) • 1 Asp94Ala (GAC-GCC) • 1 Ser91Pro (TCG-CCG) • 1 Ala90Val (GCG-GTG) • 2 wild type
RFP (1.0μg/ml)			*rpoB*	
APM-S/MYCOTB-R	4	2–4		• 1 His526Leu (CAC-CTC) • 1 His526Asp (CAC-GAC) • 1 His526Asn (CAC-AAC) • 1 DEL 509 AGCCAGCTG
Rfb (0.5μg/ml)			*rpoB*	
APM-R/MYCOTB-S	3	0.5		• 1 Ser531Leu (TCG-TTG) • 1 His526Arg (CAC-CGC) • 1 wild type
APM-S/MYCOTB-R	14	1–8		• 2 Leu533Pro (CTG-CCG) • 5 Ser531Leu (TCG-TTG) • 1 His526Tyr (CAC-TAC) • 1 Asp516Tyr (GAC-TAC) • 1 Asp516Val (GAC-GTC), His526Gln (CAC-CAG)/Leu511Pro (CTG-CCG), • 1 Asp516Glu (GAC-GAG)/Ser522Leu (TCG-TTG) • 1 514 ins TTC
EMB (5.0μg/ml)			*embB*	
APM-S/MYCOTB-R	11	8–32		• 2 Met306Ile (ATG-ATA) • 1 Met306Val (ATG-GTG) • 1 Tyr319Ser (TAT-TCT) • 1 Asp354Ala (GAC-GCC) • 6 wild type
Sm (2.0μg/ml)			*rpsL*	
APM-R/MYCOTB-S	5	1–2		• 1 Lys43Arg (AAG/AGG) • 4 wild type
APM-S/MYCOTB-R	3	4–32		• 1 Lys43Arg (AAG/AGG) • 2 wild type
Eth(5.0μg/ml)			*ethA*, *inhA*	
APM-S/MYCOTB-R	6	10–40		• 1 Arg165Gly(TGT-TGC) • 1 Ala267Gly(GCC-GGC) • 1 silent mutation: Cys137Cys(TGT-TGC)
APM-R/MYCOTB-S	1	5		• 1 C(-15)T
INH (1.0μg/ml)			*katG*, *inhA*	
APM-R/MYCOTB-S	20	0.25–1		• 8 Ser315Thr(AGC-ACC) • 1 Thr322Ala(ACG-GCG)
				• *6* C(-15)T
				• 5 wild type for both *katG* and *inhA*
APM-S/MYCOTB-R	2	4		• 2 wild type

Note: APM-S: APM susceptible; APM-R:APM resistant; MYCOTB-R:MYCOTB resistant; MYCOTB-S: MYCOTB susceptible

### Sequencing results of APM-susceptible/MYCOTB-resistant strains

65.4% (34/52) APM-S/MYCOTB-R strains had mutations or insertions in the amplicated regions in the resistance related genes. For Mfx at 0.5 μg/ml and Ofx, 5/7 (72%) and 4/5 (80%) strains had mutations in the *gyrA* gene. For RFP, 4/4 strains had mutations or nucleic acids deletion in the *rpoB* gene. For Rfb, 13/14 (93%) strains had mutations in the *rpoB* gene. 5/11 (46%) strains with APM-S/MYCOTB-R for EMB had *embB* mutation. For INH at 1.0μg/ml, two strains were both wild type for *katG* and the promoter region of *inhA*. 1/3 strains had mutation in the *rpsL* gene. 2/6 strains had mutations in the *ethA* gene.

### Sequencing results of APM-resistant/MYCOTB-susceptible strains

66.7% (20/30) APM-R/MYCOTB-S strains had mutations in the resistance related genes. For Mfx at 0.5 μg/ml, the strain had a mutation in the *gyrA* gene and the MIC was 0.5μg/ml, which was equal to the low-level critical concentration (0.5μg/ml). 2/3 strains had mutations in the *rpoB* gene and MICs for these 3 strains were all 0.5ug/ml, which were equal to Rfb critical concentration. Only 1/5 APM-R/ MYCOTB-S strains had mutations in the *rpsL* gene for Sm. The MIC range of the 5 stains was 1.0–2.0ug/ml which were well below or equal to the critical concentration. One Eth APM-R/MYCOTB-S strain occurred mutation in the promoter region of *inhA* and the MIC was equal to the critical concentration of Eth. 15/20 discordant strains for INH at 1.0μg/ml had mutations in *katG* or the promoter of *inhA* genes.

## Discussion

China has a serious epidemic of drug-resistant tuberculosis. Cheap, effective and rapid detection of drug resistance to tuberculosis is very important for patient management and TB control. The common used solid medium based proportion DST method in China was labor demanding and need at least 4–6 weeks for result interpretation. The method used one or two critical concentrations to determine qualitative susceptibility or resistance. However, some studies reported that the heterogeneous MIC levels observed in drug resistant M. tuberculosis strains may have important therapeutic implications [12,13,14,15,16]. In one study conducted by Heysell et al. showed that in response to a borderline MIC, a clinician may increase the dose of medications., such as levofloxacin or moxifloxacin, or even consider decreasing dose for drugs with toxicity, such as cycloserine, in the presence of a very low MIC[17]. So understanding of the extent of drug resistance is more meaningful for clinical treatment. The MYCOTB can not only provide the qualitative susceptibility result but also the extent of drug resistance (minimal inhibitory concentration).

In this study we showed the usefulness of the 96-well plate assay for testing of drug susceptibility of *Mycobacterium tuberculosis* in China. We did not measure turnaround time but most of MYCOTB test results were available in 10 to 14 days, which was shorter than the reporting time in agar medium (3 weeks) or L-J medium (4 -6weeks). We found that the categorical agreement between the two methods was ≥90% for eleven drugs, only not for INH at 1.0μg/ml (88.6%). The sensitivities or specificities were below 90% for Rfb (84.3%), for INH at 1.0μg/ml (84.6%) and for EMB at 10.0μg/ml (80.0%). As with previous studies[11,18], our study used the critical concentration equal to APM critical concentration as the breakpoint to interpret the MIC value on MYCOTB plate although there was also study using the 0.25μg/ml, 4.0μg/ml contained on the MYCOTB plate as breakpoints for INH, EMB for MYCOTB assay, respectively[3]. One problem using these critical concentrations equal to the APM critical concentration was that critical concentration for some drugs on APM were not contained on the MYCOTB plate, for instance, INH at 0.2μg/ml, Am at 5.0μg/ml, EMB at 5.0μg/ml and 10.0μg/ml, Sm at 10.0μg/ml and Cs at 25.0μg/ml. So the definition of “susceptibility” and “resistance” of MYCOTB by using the same critical concentration on APM may result in a disagreement where agreement may exist. To resolve this issue, as in study published by Jongseok et al[11], the “conditional agreement” was calculated, which relaxed the critical concentration to a± 1 doubling dilution around the APM critical concentration. The agreements improved for all drugs. Of note were the low categorical agreement for INH at 1.0 μg/ml (88.6%) and the low sensitivity for detection of resistance for INH at 1.0μg/ml (84.6%) for MYCOTB. Most(20/22) of discrepant strains for INH at 1.0μg/ml were resistant by APM and susceptible by MYCOTB. Among twenty strains with APM resistant and MYCOTB susceptible result, 65%(13/20) strains had MICs equal to the critical concentration of 1.0μg/ml, 6 strains had MICs of 0.5μg/ml, which was well below the CC of 1.0μg/ml and only one strain was discrepant with a MIC 0.25μg/ml. The agreement improved to 95.3% if the MIC result was relaxed to a one doubling dilution around the critical concentration for INH at 1.0μg/ml. The sequencing results of discrepant strains for INH at 1.0μg/ml showed that among 20 APM resistant and MYCOTB susceptible strains, nine strains had mutations in the *katG* gene, six strains had mutations(C-15T) in the promoter region of *inhA* gene and 5 strains were both wild type for *katG* and the promoter of *inhA* genes. For Cs the sensitivity was only 71.4%. The results in study of Jongseok et al[11] showed that the sensitivities of Cs and EMB at 10μg/ml were 18.2%(95% CI, 2.1–34.3) and 31.0% (95% CI, 19.1–42.9). The 95% confidence interval for Cs and EMB at 10μg/ml in the present study overlap those in study of Jongseok et al. Another study conducted by Laslie et al[18] reported that the sensitivities of Cs, EMB at 10ug/ml were both 100%. One study conducted in China by Yu showed that the sensitivities for Cs and EMB were 100% and 64.6%, respectively[19]. However, the number of strains resistant to Cs in the present and other three studies were all small. In the present study, sensitivity for EMB at 5μg/ml was 100% (95%CI, 90.8–100.0), similar with 98.2% in Laslie’s study, but significantly higher than that (64.3%, 95%CI (55.4–73.2)) in study of Jongseok et al^[^^11^^]^ and that in Yu’s study[19]. Some studies showed that phenotypic DST method for ethambutol is not as accurate as other drugs such as RFP or INH, with low inter-laboratory agreement[20,21]. Thus more studies based on large sample are needed to evaluate the performance of MYCOTB for testing susceptibility of these two drugs.

In the present study, most of the discrepant results for Ofx, Mfx, RFP, Rfb, Eth and EMB were APM susceptible and MYCOTB resistant. DNA sequencing of genes known to be responsible for resistance to these drugs resolved these discrepancies in favor of the MYCOTB plate for four of the above mentioned five drugs except for EMB: Ofx (4/5), Mfx (5/7), RFP (4/4) and Rfb (13/14). For EMB at 5μg/ml, nearly half (5/11) of the strains carried mutations in the *embB* gene. For Eth, 2/6 strains had mutation causing amino acid change in the *ethA* gene. MYCOTB thus showed better agreement with sequencing results than APM for Ofx, Mfx, RFP, Rfb, but not for EMB at 5μg/ml and Eth. Brossier’s study reported that only 46.8% (22/47) Eth resistant isolates mutated in *ethA*[22]. So *ethA* gene can not explain all mechanisms of Eth resistance. In Bakula’s study, sequence analysis of the EMB resistance determining region (ERDR) in the *embB* gene was not sufficient for rapid detection of EMB resistance, and mutations in codon 306 were not good markers of resistance to EMB[23]. A study conducted by Zhang et al[24] showed that the concordance rate for EMB between the MIC broth method and sequencing was significantly higher than that between the L-J medium (2μg/ml) method and sequencing. For Cs there were no obvious gene mutations conferring drug resistance. For PAS, association between mutations in *thyA* and PAS resistance was limited [25], so we did not sequenced the genes to investigate the discrepancies at molecular level for Cs and PAS. 5/10 discrepant strains with APM-R/MYCOTB-S result for Mfx, Rfb, Sm and Eth had mutations in the resistance related genes. Of note, MICs of all 10 isolates with discordant results characterized as APM-R/MYCOTB-S were equal to or within 1 doubling dilution of the critical concentration, which made interpretation for these strains difficult.

In the present study, we used APM as reference method. The reasons that we choose APM method but not MGIT method as reference were as following, 1) critical concentrations for Rfb and Cs for MGIT method were not recommend by WHO. 2) APM method contained two critical concentrations for both low level and high level resistance for INH, EMB, Mfx and Sm. 3) When the critical concentration differed between the APM breakpoint and the MGIT 960 breakpoint, the accuracy was superior when the APM breakpoint was used for all drugs except for INH and PAS as Heysell et al reported[5]. Other conventional phenotypic methods such as MGIT960 and L-J medium proportion had some limitations. Sayera et al found that discrepancies between MGIT960, L-J method, MYCOTB, quantity PCR for EMB were mainly from MGIT susceptible and resistant by other methods and for Sm were mainly due to false resistant by L-J proportion method[4].

There are some limitations for the present study. The first limitation was that the numbers of strains resistant to Am, Km and especially Cs were low (less than 10) resulting to low precision for the sensitivity calculation, and so further validation study was necessary for these drugs. The second limitation was that the mechanisms of resistance for Cs and PAS were not clear and the genotypic or other phenotypic methods were not conducted to resolve the discrepancy between APM and MYCOTB assay. The last one was that we did not inoculate the strain suspensions on blood agar for exclusion of contamination. The step of subculture on blood agar to check for fast growing non-tuberculosis mycobacteria and/or bacterial/fungal contamination was necessary for MYCOTB assay. In the present study, the preserved pure Mycobacterial tuberculosis strains but not clinical strains were used for the assessment and we therefore roughly checked the plate for contamination after 24 and 48 hours of incubation. If the MYCOTB assay will be used for clinical diagnosis, the step of inoculation suspensions on blood agar for exclusion of contamination is absolutely essential.

Nucleid acid amplication based Genotype MTBDR*sl* at the moment is the only real rapid test for second-line drugs. However, MTBDR*sl* can not replace conventional drug susceptibility due to the low sensitivities for detection of resistance of kanamycin (42.7%-43.1%), capreomycin (71.4%-90.6%) and ethambutol (56.2%-77.3%) and the limited number of drugs it tests for[26,27,28]. It is only recommend to be used as a rule-in test for additional resistance to fluoroquinolines and aminoglycosides for MDR-TB patients. The MYCOTB is easily performed during inoculation, incubation and reading especially using the automatic inoculation instrument and plate reader. There was a good agreement between MYCOTB and APM for most of drugs tested, and the assay can be custom-made by manufacturer to determine drug resistance to drugs most relevant to the local drug resistance profile and treatment regimens based on the requirement of local area. Therefore, it could be used as an alternative to the more labor demanding solid culture based DST method. A strategy for combining the use of the MYCOTB plate and molecular assay could be using molecular assay as initial rapid diagnostic testing for identifying MDR cases and using MYCOTB plate as subsequent testing to screen other first-line and second line drug resistance to provide the best possible treatment regimen especially in low-resource settings. However, more large sample studies are needed to further evaluate the performance of MYCOTB in clinical drug resistance diagnosis.
